# Health marketing and behavioral change: a review of the literature

**Published:** 2018

**Authors:** Cristina-Mihaela Chichirez, Victor Lorin Purcărea

**Affiliations:** * “Carol Davila” University of Medicine and Pharmacy, Bucharest, Romania

**Keywords:** behavioral change, health marketing, lifestyle, social marketing

## Abstract

Health marketing as a part of social marketing, must influence individuals, voluntarily, through various social programmes, in order to accept, reject, modify or abandon a behavior in favour of a healthier lifestyle.

Acting on individual behavior change, social marketing can influence the behaviour of those who decide public policies, with positive effects in social change. In time, in order to understand and predict a behavior, a number of theories, models and tactics were developed with the aim to identify factors and mechanisms with the greatest impact in the changing process. Cognitive- social theories proved to be more effective, because they offer guidelines for conducting research in behavioral change.

Health marketing is the component of Social Marketing which uses commercial marketing principles and techniques to influence a target audience, so as, on a voluntary basis, individuals may accept, reject, modify or abandon a behavior for their own benefit , groups or for society as a whole [**1**]. Moreover, it is assumed that social marketing is not a new concept, it even ocurred before commercial marketing. In U.S.A., if for commercial marketing, its beginnings are considered to be around 1900 [**2**], the social marketing principles have come forward since the second half of the 19th century, as for example, in the abolition of slavery in 1862, then in the right of women's suffrage and the abolition of child labor in 1869 [**3**].

The concept of social marketing has been introduced for the first time, in the academic literature, by Philip Kotler and Gerald Zaltman, in Journal of Marketing. Social Marketing describes ʺuse marketing principles and techniques to identify a social issue, idea or behavior" [**4**].

In recent years, the interest in social marketing has increased, with a special interest in social issues related to public health, prevention, environment, community development and welfare [**3**]. Also, MacFadyen and collaborators state that social marketing provides the necessary tools to address these problems by influencing individuals with the purpose of adopting new behaviors and healthier lifestyles [**5**]. For instance, social marketers had as main objective in U.S.A. influencing the behavior of volunteer individuals in relation to reducing obesity, smoking cessation, encouraging walking, cycling and in Africa, the focus moved to solving some major health problems, such as the prevention of malaria, poliomyelitis eradication, decreasing infant mortality, stopping the spread of HIV/AIDS.

At the same time, social marketing can influence the behavior of those who decide public policies and of those belonging to various interest groups, with positive effects in social change, including law, public policy, and community involvement, educational curriculum, business practices, and others [**3**].

Rob Donovan and Nadine Henley mention the fact that influencing individual behavior has the power to determine a behavior change of those people who can facilitate other behaviors and can may elaborate on institutional policies and legislative changes in the social structure [**6**].

Although there are similar principles and techniques implemented, between commercial and social marketing there are a number of differences. Thus, while commercial marketing is aimed at the sale of goods and services, which bring profits for the organization, social marketing seeks to sell a change in behavior for the benefit of the individual and society.

In their work entitled "Social Marketing. A synopsis by the Center for Social Marketing ", MacFadyen, Stead and Hastings highlight the types of behavioral change produced by the social marketing . So, on the short term, at the individual level may take place behavioral changes, at the level of the group of individuals may be registered changes in the rules and administrative policies and at the level of society, may be registered changes in public policies, respectively [**5**].

On the long term, the level of the individual changes occurs in lifestyle, at the level of the group of individuals, changes may occur in organizations and at the level of society, change may take place a social and cultural environment. Also, specialists consider that social marketing is much more complex than commercial marketing regarding the following aspects: - the products of social marketing are much more diverse; - the application of products is different from individual to individual; - target groups are much more difficult to attain; - the involvement of the consumer is much higher; - the competition is much more subtle and more varied.

A major feature of Social Marketing is that of recognizing positive behaviors more than the punishment of the negative ones by forms of influencing economic or compelling.

Social marketers can not promise effects and immediate benefits in exchange for the adoption of the proposed behavior, but they may determine the target audience to take action in the following directions [**3**]: - to continue with the healthy habits; - to accept a new behavior; - to reject a potential undesirable behavior; - to modify a current behavior; - to abandon one of the old undesirable behavior.

Some experts consider that along time, the financial resources of individuals have made unhealthy habits to become normal and therefore it is necessary to support behavioral changes with respect to modifying their lifestyles, stopping unhealthy habits, by establishment of new habits, build physiological resistance to their unhealthy needs.

Due to the fact that the negative effects of the behaviors on health status are more visible, a series of policies and strategies have been drawn up to change them. At the same time, it should be taken in consideration that health has multiple determinants, of genetic origin, psychological, social, economic factors which interact and determine determining their outcome the behavior of the individual [**7**].

In their work "Theory and Research in Promoting Public Health", Wills and Earle identify three levels at which behavioral change may operate: at the biomedical level, behavioral level and at the social level [**8**].

Because behaviors are extremely complex, the specialists have drawn up a series of theories, models and tactics with regard to the understanding and prediction of behavior, as an alternative to the model of the biomedical device which dominates public health. According to this concept, disease is explained by the cause-effect relationship, stimulus-feedback, while the operations to change behavior is based on the measurement of the attitudes, the perceptions of the target group [**9**].

Theories, models and tactics presented in the scientific literature, can identify factors and mechanisms with the highest relevance to a specific behavior and provide interventional strategies and programs which may trigger [**10**].

Theories are designed to simplify the elements and phenomena encountered in the environment, giving them an intelligible form [**11**], and it is necessary to know the factors which affect them, the relations between variables and the circumstances in which these relationships occur or not [**12**].

Consequently, they are made up of a series of interrelated concepts and definitions which gives an overview of events and situations, thus facilitating their understanding by specifying the relations established among variables [**13**]. Models are descriptions based on hypothetical analogy, foremost, explaining in detail the phenomenon, and in fact, being part of the theory [**14**].

Moreover, the concept is the basic element of a theory, and the operational form of the concept is called variable. In theory, many associated concepts form a construct [**15**].

Behavioral theories were divided into continuous theories and stadial theories [**16**]. Continuous theories are based on the identification of variables that influence action and work under several combinations which produce a single prediction outcome in the shape of an equation for all individuals [**17**]. Stadial theories involve multiple stages and a number of conditions by which some individuals may be motivated enough to move from one stage to the other [**18**].

From all continuous major theories, the most important theories are those which offer social, cognitive, and theoretical framework for research and in addition select variables involved in the health and development of the most effective prediction of the individual's motivation for interventions in adopting a certain behavior [**19**].

The most important behavioral change model is Bandura`s called Social Cognitive Theory, based on the principles of social learning, which state that children’s and adults' social-cognitive experiences are reflected in their behaviors [**20**]. The theory was first developed by Rotter [**21**], known as "Social learning theories” that rely on stimulus-response theory. Bandura as well as social-cognitive theory advocates argue that impulse received from the feedback of an individual is not enough to explain the whole human behavior. Thus, in his work "Social Foundation of thought and action of social-cognitive theory", Albert Bandura defined behavior as the result of interaction between the personal factors (personal effectiveness), and environmental action [**22**]. In 2001, Bandura mentioned that "internal factors of personal nature in form of cognitive, affective and biological events, behaviors patterns and environmental influences operate as factors that are influencing each other" [**23**].

Personal effectiveness is considered to be the strongest predictor of behavior and is used in Behavioral Research studies and in health education for health [**24**].

Further, personal effectiveness expresses a person's confidence in his requested abilities for the adoption of a new behavior through actions concerning the consequences of the situation, expectations, social and environmental norms.

Motivation and action are governed by forward-looking thoughts that include expectations regarding the consequences of the situation, expectations regarding the action and efficacy expectations. To perform a specific behavior, first individuals must know it and their ability to meet a series of behaviors is called "behavioral capacity".

In 1967, in his work, "A behavior theory approach to the relation between beliefs about an object and attitude toward the object", Martin Fishbein, developed the Theory of Reasoned Action, suggesting that attitudes influence behavior and that the best predictor of a person's behavior is the intention to act [**25**].

Together with Icek Ajzen in the work "Belief, attitude, intention and behavior: An introduction to theory and research" (1975), he developed a structure for the analysis of the interaction between the attitudes, beliefs and behavioral intention [**26**]. However, they concluded that the behavior is more influenced by the so-called subjective rules, namely the individual's perception of how others relate to the behavior in question [**27**].

Consequently, in 1988, Ajzen expanded and revised the Theory of Reasoned Action by adding a new predictor variable called the Rerceived Behavioural Control, resulting in "Theory of Planned Behavior". This addition takes into account the fact that the individual wishes to adopt a new behavior when he has a positive attitude towards a situation and he perceives that situation to be important and beneficial for him and believe that he would be successful in adopting that behaviour. Thus, theory is based on cognitive behavior, without taking into account emotional variables [**3**]. Ajzen justified the importance of this predictor in the individual's motivation, explaining that if there is no perceived behavioral control, the attitude towards the behavior and subjective norms are not capable to generate a sufficient motivation, to trigger an intention [**28**].

In the late 1950s, Paulo Freire, a Brazilian teacher who wrote 20 books about pedagogy and education, initiated a program of education for the residents of the slums and the people living from rural areas, putting the basis for "Liberation Theory “[**29**].

The theory was later developed by Greenberg (1978) and Wallerstein and Bernstein (1988), focusing on education and empowerment (freedom of informed decision making).

In his work "How does a language acquire gender markers", Greenberg (1978) mentioned that health education has the role to " determine people to make informed decisions about their health, based on their own needs and interests, as long as their decisions not affect the needs and interests of other members in the society."[**30**].

In the work "Empowerment education - Freire's ideas adapted to health education", Wallernstein and Bernstein emphasized personal empowerment, defining it as "a social process that promotes the participation of individuals, organizations and communities in the exercise control over their own lives and the community in general. From this point of view, empowerment is not the control exercised over other people's behavior, but rather the ability to act with other members of society to make a changeʺ [**31**]. Also, Wallernstein and Bernstein mention that three different stages which individuals need to follow along the changing process.

In the first stage, the information from the target population is gathered, the needs of the community are prioritized and an action plan is drawn up. The difference is methodology meaning that information is not collected and prioritized by marketers, but by the target population. The second stage follows the education itself, based on a dialogue between marketers and the target population, marketers helping participants to elaborate their problems and action plans. In the third stage, the plans of the target population are implemented, followed by their monitoring, re-evaluation and review.

This theory has the advantage of responding directly to the needs of the community, identifying issues by needs. A disadvantage is the fact that large groups of people are working, which makes coordination more difficult.

The model of trust in the health has been developed by the social psychologists Godfrey H. Hochbaum [**32**], Irwin M. Rosenstock [**33**] and Victor J. Stretcher [**34**] and is a systematic method to explain and predict the preventive behaviors, in fact these specialist improved the work of Kurt Lewin [**35**].

Kurt Lewin proposed an approach of change by analysing the opposite direction forces, namely, generating change forces and forces resistante to change. He described the change in humans by using to the shape of an ice cube which consists of a several status changes such as the melting of ice, passing through the liquid phase and freezing water in a different form.

Lewin considered that the change has must imposed and the resistance to change can be managed by setting out the objectives at the beginning of the process which define the direction of evolution.

In the beginning, the model of trust in health has been used to understand the preventive behaviors, afterwards it was used to differentiate between a favorable behavior and a conduct harmful to health, respectively for the understanding of the related psycho-social behavior determinants of the favorable or unfavorable health, which includes six variables [**36**][**37**]: - perceived susceptibility (the perception affecting health is conditioned by a certain fact); - prediction of the severity of the consequences (the belief of a person that the effect of a disease or a status will pass if he acts ); - the benefits of the adopted behavior; - the costs and barriers perceived in action (the existence of a reason for why the behavior could be perceived as incompatible, expensive, painful, unpleasant or unstable); - the motivation to act ( internal or external strategies which might be required for the adoption of the behavior); - the motivation for health (the individual must benefit from a series of impulses to convince him to change).

According to this model, once an individual perceives a threat to his health and the simultaneous presence of the action for the assessment of the healthy behavior, brings an advantage, he will act to favorable change favorable.

Another model concerning behavioral change is the Transtheoretic Model elaborated in 1979 by James D. Prochaska [**38**] and then improved it in 1982 together with Carlo C. DiClemente [**39**]. The development of the model is based on the principle of identifying the stage in which an individual is, each stage being defined by his previous behavior and future plans.

In the work "Changing for good", Prochaska and collaborators [**40**] describe the six stages of behavioral change, and eventually remove the last, as follows: 1. precontemplation (the individual does not intend to change his behavior and do not recognize that he has an issue); 2. contemplation (the individual realizes that he has a problem and begins to think about it, accepting the opportunity to make a change in his behavior); 3. preparation (the individual plans his actions with a perspective on adopting a new behavior); 4. action (the decision to change has been taken and the individual operates in the direction of adopting the new behavior); 5. maintaining (actions are directed towards the stabilization of the new behavior and the prevention of bad behavior); 6. completion (new behavior is strengthened and there is no longer a danger to return to the old habits).

In their work "Stages theories of health behavior: The conceptual and methodological issues", Weinstein and collaborators (1998) identified four characteristics of the stages of changing behavior: - the stage description process; - the stages must be arranged in a certain order; - in the same stage, obstacles should be common; - in different stages, the obstacles may be different.

The transtheoretic model aims at adopting healthy behavior or giving up a bad behavior as a result of a singular, rational and perfectly conscious decision of the individual. It was originally designed for the treatment of the drugs and alcohol, but then it has been checked and on other behavioral patterns in education and health.

These theories and models have a special importance for social marketers, helping them to identify the key and intervention mechanisms in the social marketing programs.

Due to the high degree of complexity which behavioral change implies, generally, one theory is often insufficient, and there are several theories which need to be synthesized in order to determine a strategic pattern to have the desired outcomes.

## Conflict of interest

The authors declare no conflict of interest.
